# Advances in the application of PD-1/PD-L1 immunotherapy for prostate cancer: a review

**DOI:** 10.3389/fimmu.2025.1664587

**Published:** 2025-12-19

**Authors:** Guangqiang Hu, Zeng Li, Yongji Chen, Hong Liao, Shukui Zhou

**Affiliations:** 1 Department of Urology, Sichuan Clinical Research Center for Cancer, Sichuan Cancer Hospital and Institute, Sichuan Cancer Center, School of Medicine, University of Electronic Science and Technology of China, Chengdu, China; 2 Department of Urology, Sichuan Clinical Research Center for Cancer, Sichuan Cancer Hospital and Institute, Sichuan Cancer Center, University of Electronic Science and Technology of China, Chengdu, China

**Keywords:** prostate cancer, PD-1/PD-L1, immune microenvironment, immunotherapy, combined therapy

## Abstract

Prostate cancer (PCa), the most prevalent malignancy among male genitourinary cancers, is distinguished by its immunologically ”cold” phenotype with persistently high incidence and mortality. Radical prostatectomy and radiotherapy represent the current standard of care for localized prostate cancer. However, recurrence or progression occurs frequently, and advanced or metastatic disease is common at initial presentation. Recent progress in cancer immunotherapy reveals that modulation of immune responses represents an effective strategy for enhancing antitumor activity. Programmed Cell Death Protein 1 and Programmed Cell Death Ligand 1 (PD-1/PD-L1) inhibitors, which restore T-cell function and remodel the tumor immune microenvironment, have achieved clinical success in melanoma, lymphoma and non-small-cell lung cancer. Although their efficacy as monotherapy in PCa remains limited and optimal patient selection criteria are lacking, emerging evidence suggests that combination immunotherapy regimens may offer clinically significant benefits. This review critically evaluates current clinical trial outcomes involving PD-1/PD-L1 inhibitors for prostate cancer and outlines priority directions for future investigation.

## 1 Introduction

**Figure 1:**
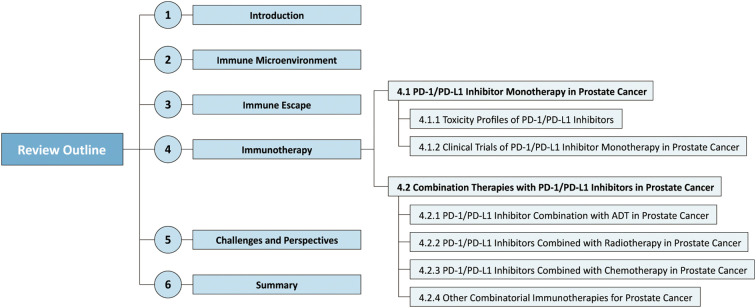
Overview diagram of the review. This paper is structured as follows: the immune microenvironment, immune escape mechanisms, PD-1/PD-L1-targeted immunotherapy, clinical challenges, and therapeutic prospects in prostate cancer.

Prostate cancer (PCa) ‌continues to represent both the most frequently diagnosed cancer and the second primary cause of cancer-specific death‌ among males in Europe and the United States (1, 2). Global epidemiological trends demonstrate a marked increase in PCa burden, with cases (+116.11%), deaths (+108.94%), and disability-adjusted life years (+98.25%) all nearly doubling between 1990 and 2019 (3). Projected annual incidence shows 299,010 cases (29% of male cancers) alongside 35,250 deaths (the third among male cancer deaths) in 2024 (2). By 2025 (4), these estimates will climb to 313,780 cases (30%) and 35,770 deaths. Current trajectories suggest global annual incidence will surpass 2.9 million cases by 2040 (5), underscoring a critical public health concern. Standard treatments for advanced PCa—androgen deprivation therapy (ADT) (6), radiotherapy (7), and chemotherapy (8)—frequently fail due to rapid drug resistance and limited long-term survival improvements. Immune evasion is promoted by immunosuppressive signals transmitted throught the PD-1/PD-L1 axis, a pivotal immune checkpoint. Although PD-1/PD-L1 inhibitors demonstrate clinical efficacy in certain solid tumors, their utility in PCa remains unclear. This review consolidates existing findings on the immune microenvironment, immune escape mechanisms, PD-1/PD-L1-targeted immunotherapy, and clinical implementation challenges (**Figure 1**). The goal is to evaluate therapeutic limitations and explore future research opportunities in cancer immunotherapy.

## 2 The immune microenvironment within prostate cancer tumors

PCa incidence ‌increases at an annual rate of 3%‌, ‌making it the most prevalent cancer among men worldwide, with‌ nearly half of cases ‌diagnosed at advanced stages (2, 9, 10). A distinct tumor microenvironment is a notable feature observed across multiple forms of PCa. The immune scoring system classifies solid tumors as either immunologically active "hot tumors"—such as melanoma, lymphoma, and liver cancer—or as immune-privileged "cold tumors," based on an evaluation of tumor-infiltrating lymphocyte density, functional status, and inflammatory characteristics within both the tumor core and the invasive margin (11, 12). PCa exemplifies a heterogeneous “cold tumor”, exhibiting minimal T-cell infiltration and low tumor mutation burden (TMB) (13). Primary lesions show mutation rates below 1mut/Mb, contrasting with metastatic lesions that may reach 4mut/Mb (14, 15). This limited TMB reduces neoantigen generation, leaving PCa devoid of potent immunogenic targets. The PCa tumor microenvironment contains immune effector cells such as CD8+ cytotoxic T lymphocytes, natural killer (NK) cells, neutrophils, B cells, and proinflammatory M1 macrophages (16), but immunosuppressive populations like myeloid-derived suppressor cells (MDSCs) and regulatory T cells (Tregs) predominate (17, 18). ‌Immunosuppressive cytokines—notably IL-10, IL-35, and TGF-β—directly inhibit T-cell activation and cytotoxic function (19).

MDSCs constitute a heterogeneous cell population, typically categorized into polymorphonuclear (PMD-MDSCs) and monocytic (M-MDSCs) subtypes, which exhibit potent immunosuppressive capabilities. These cells can suppress NK cells, macrophages, and effector T cells, while also promoting the expansion of Tregs and enhancing their immunosuppressive function (20–22).Tregs, a subset of CD4se cells with immunosuppressive functions, facilitate tumor immune evasion by directly suppressing effector T cell activity, secreting immunosuppressive cytokines such as IL-10, reducing T cell metabolic activity, and inhibiting antigen-presenting cell function through contact-dependent mechanisms (22–24). Clinical evidence (25–29) indicates that MDSCs and Tregs are enriched in advanced PCa tissues and are associated with disease progression and poor prognosis. MDSCs and Tregs form a stable “immunosuppressive hub” through chemokine-mediated recruitment and functional cross-reinforcement. For instance, MDSCs secrete chemokine ligand 22 (CCL22), which recruits Tregs to the tumor microenvironment by binding to chemokine receptor 4 (CCR4) on Treg surfaces (21). Transforming growth factor-β (TGF-β) secreted by Tregs upregulates the expression of arginase-1 (Arg-1) and indoleamine 2,3-dioxygenase (IDO) in MDSCs by activating the Smad3 pathway. Both Arg-1 and IDO inhibit T cell receptor expression and induce T cell apoptosis via their metabolites. In co-culture experiments, Treg-conditioned medium enhanced Arg-1 and IDO activity in MDSCs, an effect that could be blocked by TGF-β neutralizing antibodies (22). Further analysis of clinical samples revealed that patients with co-enrichment of MDSCs and Tregs had a significantly lower response rate to docetaxel combined with ADT compared to those without co-enrichment (18% vs. 45%) (30).

Single-cell transcriptome analyses demonstrate reduced expression of T-cell cytotoxicity-related genes alongside marked elevation of exhaustion markers (e.g., TIM-3 and PD-1) in PCa tissues relative to melanoma (31). Tumor cells further impair T-cell transendothelial migration by suppressing adhesion molecules such as ICAM-1 and VCAM-1 (32). Patients with metastatic castration-resistant prostate cancer (mCRPC) undergoing novel endocrine therapies (abiraterone or enzalutamide) show decreased peripheral blood T-cell counts (33), while exhibiting elevated circulating proportions of Tregs, M2-polarized macrophages, and MDSCs (34, 35).

In the immune microenvironment of PCa, MDSCs and Tregs form a synergistic immunosuppressive network through a triple interaction of “chemoattractants-cytokines-metabolites”: On one hand, TGF-β, secreted by tumor cells and Tregs binds to TGF-βRII on the surface of MDSCs, activating Smad2/3 phosphorylation and upregulating the expression of Arg-1 and CCL22, thereby enhancing the metabolic suppression mediated by MDSCs (36). TGF-β can also promote the differentiation of naive CD4+ T cells into Foxp3+ Tregs via a Smad4-dependent pathway (22). On the other hand, IL-23 secreted by MDSCs induces the differentiation of CD4+ T cells into Th17 cells by activating the STAT3 pathway; IL-17 secreted by Th17 cells binds to IL-17R on the surface of Tregs, activating the NF-κB pathway and upregulating the expression of Foxp3 and CTLA-4, thereby maintaining Treg stability and enhancing their function (30, 37). Additionally, MDSCs and Tregs can cooperatively generate adenosine through CD39/CD73, collectively suppressing CD8+ T cells (37).

PCa establishes a unique TME through diverse genomic mechanisms: mCRPC tissues frequently carry somatic or germline mutations in DNA repair genes, such as BRCA1/2 alterations, CDK12 inactivation, and RB1 shallow deletions, with homologous recombination defects occurring in 20%–25% (38). PTEN and TP53 aberrations appear in 25% of primary tumors and 60% of mCRPC cases (14, 15, 39, 40). CDK12 is a key gene for DNA repair, which is involved in cell cycle progression and transcription elongation (41). Loss-of-function mutations in this gene are often characterized by increased focal tandem duplications, elevated gene fusion events, and higher tumor neoantigen burden (42).

Functionally, NK cells in prostate cancer patients exhibit suppression and exhaustion, characterized by the expression of markers such as PD-1 and TIM-3 and the downregulation of activation receptors like NKG2D; this leads to a reduction in both cytotoxic activity and cytokine secretion (43, 44). Hypoxia, acidity, and metabolic waste accumulation in the TME can trigger NK cell dysfunction; TGF-β secreted by tumor cells can downregulate the expression of NK cell activation receptors, thereby impairing their ability to recognize tumor cells (45, 46). Additionally, PD-1 binding to PD-L1 inhibits both NK cell degranulation and IFN-γ secretion, activates SHP phosphatase, and suppresses phosphorylation within the PI3K–AKT pathway, thereby blocking the cytotoxic function of NK cells (47).Blocking PD-L1 enhances the anti-tumor activity of NK cells and increases tumor clearance rates (48), although some models rely on T cell cooperation. In the PD-L1 high-expressing lung cancer cell line H441 (49), avelumab combined with NK cells induced approximately 46.4% antibody-dependent cellular cytotoxicity (ADCC) lysis. Simultaneously blocking the PD-L1 and TGF-1 pathways (50) further enhances immune cell infiltration; the dual-target inhibitor M7824, at doses exceeding 1 mg/kg, fully occupies PD-L1 receptors and neutralizes all TGF-β isoforms (TGF-β1, β2, and β3) in plasma. Combined blockade of B7-H3 and PD-L1 (10), elimination of MDSCs in the tumor microenvironment (46), and blockade of TIGIT (51) (T cell immunoglobulin and ITIM domain protein) can all partially restore NK cell activity and function.

Dysregulated AR signaling, including AR amplification and AR-V7 expression, persists under androgen-deprived conditions; AR-V7 not only constitutively activates AR signaling pathways but also suppresses antigen presentation, thereby reinforcing an immunosuppressive state (52). AR-V7, a splice variant of the AR, constitutively activates AR signaling under low androgen conditions or in the presence of AR antagonists. It can also bind directly to androgen response elements in the PD-L1 promoter and enhance PD-L1 transcription (53–55). AR-V7 blockade (55) suppresses prostate tumor growth in mice and eradicates intraosseous tumor cells. Clinical evidence (52) indicates that although nivolumab plus ipilimumab shows limited overall efficacy in unselected AR-V7-positive mCRPC, a subset of patients (23.3%) achieved sustained progression-free survival exceeding 24 weeks, suggesting a potentially responsive subgroup. This effect may be attributable to DNA repair deficiencies that elevate tumor mutational burden and thereby potentiate responses to immunotherapy. These interconnected mechanisms perpetuate an immune-hormonal vicious cycle.

**Figure 2:**
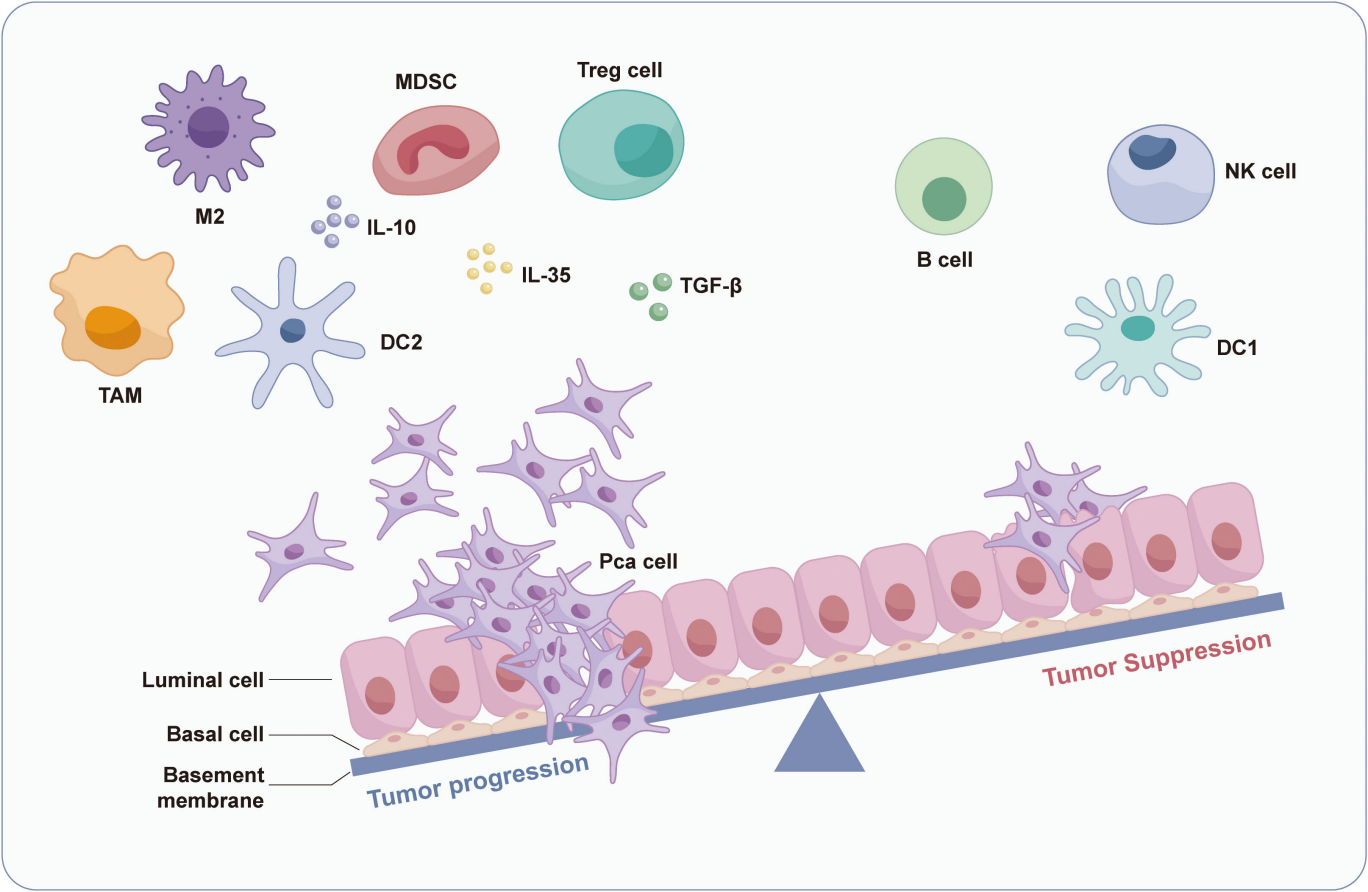
Prostate cancer immune microenvironment. This figure illustrates the distinct tumor microenvironment in prostate cancer. Despite the presence of immune effector cells, immunosuppressive cells predominate and coexist with elevated levels of immunosuppressive cytokines. This milieu facilitates tumor immune evasion, ultimately culminating in disease progression.

Metastatic lesion single-cell transcriptome analysis (56) demonstrates increased CD8+ T lymphocyte, NK cell, and monocyte infiltration in metastases relative to primary tumors, suggesting compensatory immune activation. Despite this response, downregulated Major Histocompatibility Complex I molecules (MHC I) expression and impaired interferon signaling pathways—including defective STAT1 phosphorylation (57, 58)—promote immune evasion, immunosuppression, and tumor immune inertia in PCa (**Figure 2**), characteristic of a “cold tumor” phenotype.

## 3 The PD-1/PD-L1 checkpoint and immune suppression in prostate cancer

The limited efficacy of PD-1/PD-L1 blockade in PCa – despite the immune checkpoint inhibitors (ICI) therapy’s transformative impact on solid tumors – highlights the imperative to delineate PCa’s distinct immune evasion mechanisms. The PD-1/PD-L1 checkpoint axis transduces immunosuppressive signals that compromise antitumor immunity. Structurally, PD-1 possesses: (i) an extracellular IgV-like ligand-binding domain, (ii) a transmembrane region, and (iii) an intracellular domain with an embedded immunoreceptor tyrosine-based inhibitory motif (59). This receptor regulates T-cell function by modulating apoptosis and acts as a pivotal negative immune regulator (60). Tissue-specific PD-1 expression patterns reveal abundant presence in peripheral memory T cells but negligible levels in monocytes, dendritic cells, NK cells and B cells (59, 61, 62). Under normal physiological conditions, this system preserves immune homeostasis by curbing excessive activation (63). Phosphorylated tyrosine residues within the PD-1 intracellular domain function as docking sites that recruit SHP-1 and SHP-2 phosphatases (64).

Within the TME, PD-L1 serves as the predominant immunosuppressive ligand, constitutively expressed on both malignant cells and key immune populations including dendritic cells, macrophages, and T lymphocytes (65). The PD-L1 topology includes extracellular IgV and IgC domains (responsible for PD-1 engagement), a transmembrane helix, and a truncated cytoplasmic tail without known signaling functionality (66). The recruitment of SHP-2 by ligated PD-1 disrupts proximal TCR and CD28 signaling, thereby inhibiting T-cell effector functions including proliferation, cytotoxicity, and cytokine secretion (67–69).

In PCa, epigenetic modifiers and signal transducers can regulate the expression of PD-1/PD-L1 through multi-level pathways, shape the immune microenvironment, and thereby promote tumor immune escape. Specifically: p300/CBP can directly activate PD-L1 transcription through H3K27ac (70); IL30 indirectly upregulates PD-L1 expression through the STAT1/STAT3 pathway (71); histone deacetylases (72) and ARID1A (the core subunit of the SWI/SNF complex) (73) inhibit PD-L1 expression through negative regulatory pathways (when they are deficient, the tumor’s immune escape ability is significantly enhanced); DNA methylation (74) (e.g., GSTP1, i.e., glutathione S-transferase Pi1, whose promoter is methylated; and RARβ2, i.e., retinoic acid receptor β2, whose promoter is methylated) can indirectly upregulate PD-L1 expression through the NF-κB and Wnt/β-catenin signaling pathways, respectively; EZH2 (histone methyltransferase) inhibitors (75) can activate the dsRNA-STING-IFN pathway, increase the secretion level of IFN-γ and thereby induce PD-L1 expression. In addition, it is worth noting that the ketogenic diet (76) can also upregulate PD-L1 expression to a certain extent.

The PD-1/PD-L1 checkpoint axis mediates immune evasion to facilitate PCa progression. In *Pten* conditional knockout mouse models of PCa (77), compared to IL-17RC knockout and lean controls, both IL-17RC wild-type and obese mice demonstrated substantial co-upregulation of PD-1 and PD-L1 in prostate tumor tissues. These findings imply that PD-1/PD-L1 pathway activation may contribute to tumor aggressiveness and immune resistance in PCa. Notably, granulocyte-macrophage colony-stimulating factor vaccination that blocked PD-1 activated CD8+ T cells but failed to eliminate tumors completely, coinciding with increased PD-L1 expression (78). This demonstrates how tumors adaptively resist immune surveillance by upregulating PD-L1. Clinical evidence further links PD-L1 expression to disease progression, as dendritic cells from mCRPC patients showed elevated PD-L1 levels (79). Analysis of mCRPC patients circulating tumor cells (CTCs) (80) revealed PD-L1 protein in over 50% of CTCs, PD-L1 mRNA overexpression in 48% of patients, and both PD-L1-positive and -negative CTCs in 37.5% of cases, highlighting tumor heterogeneity. Longitudinal data (81) revealed that PD-L1 positivity in CTCs increased from 40% at initial diagnosis to 70% after treatment as PCa transitioned from hormone-sensitive to castration-resistant stages, mirroring disease aggressiveness. Histological examination of 220 radical prostatectomy specimens demonstrated PD-1 and PD-L1 expression in only 1.5% and 0.5% of benign tissues, respectively, compared to 7.7% and 13.2% in tumor tissues (82). Among mCRPC cases, PD-L1 expression reached 32.1% (83). While methodological differences and tumor heterogeneity account for some variability across studies, the consensus confirms progressive PD-1/PD-L1 upregulation during PCa advancement (84, 85).

**Figure 3:**
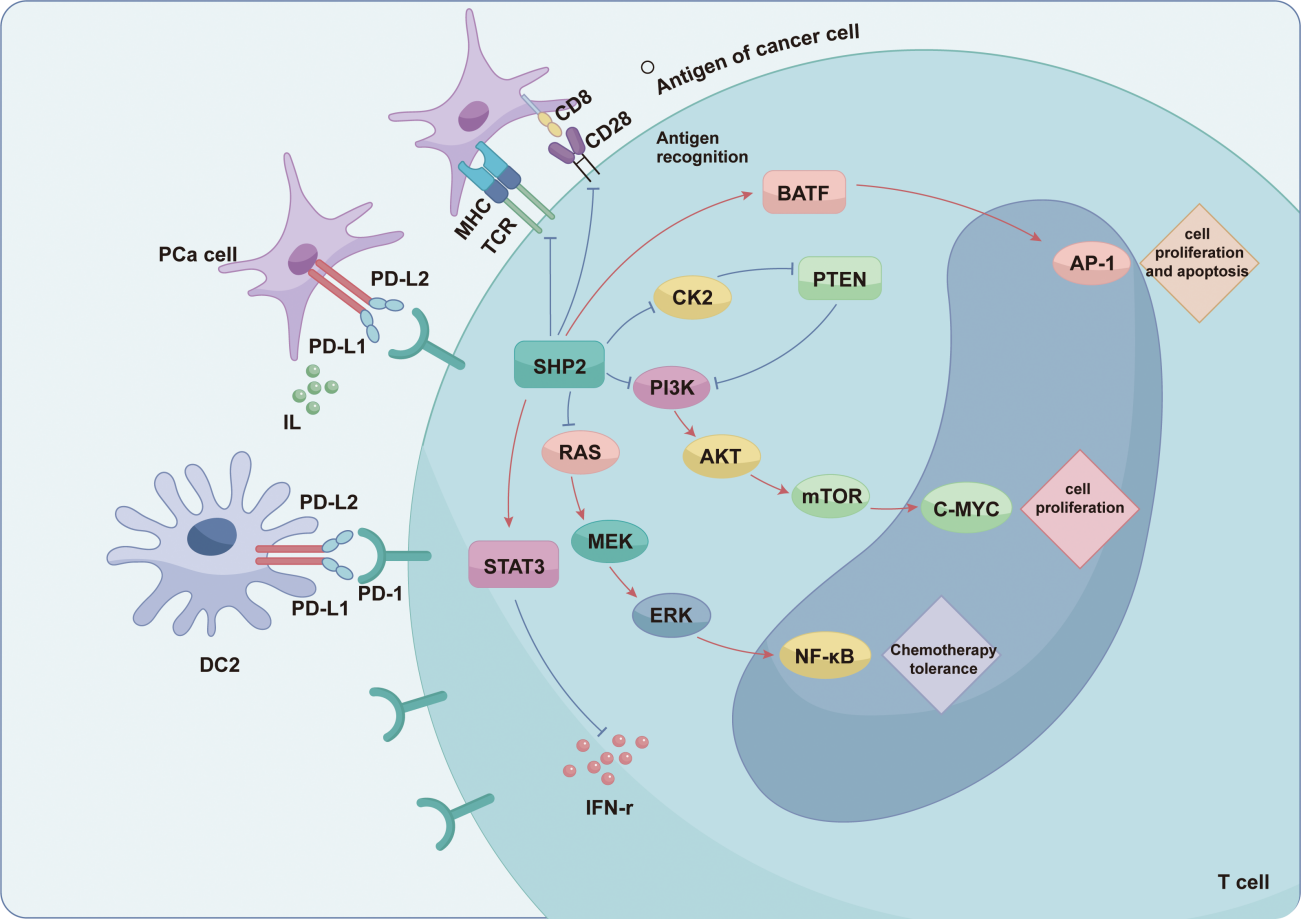
PD-1/PD-L1 checkpoint-mediated immunosuppressive mechanism. Prostate cancer cells subvert antitumor immunity by targeting the PD-1/PD-L1 axis, thereby impairing TCR-CD28 co-stimulatory interactions. This disruption dysregulates critical signaling pathways—including PI3K/AKT and RAS-MEK-ERK cascades—while suppressing secretion of effector molecules such as IFN-γ. Consequently, T cells exhibit restricted proliferative capacity, functional exhaustion, and acquired chemoresistance.

PCa cells hijack the PD-1/PD-L1 axis to orchestrate a multifaceted immunosuppressive network enabling immune evasion (**Figure 3**). A key mechanism involves the phosphorylation of PD-1’s immunoreceptor tyrosine-based switch motif, which facilitates SHP-1/SHP-2 binding and consequent dampening of antitumor T-cell signaling (86). This cascade impairs T-cell function through multiple mechanisms: it disrupts TCR–pMHC–CD8 interactions and thereby compromises antigen recognition (87); activates PTEN to suppress the TCR-mediated PI3K/AKT pathway, limiting T-cell proliferation (88); amplifies STAT3-mediated negative feedback, which reduces NK cell responsiveness (89); and suppresses PKCδ activation, diminishing effector molecule production (90).Consequently, T cells within the TME become functionally impaired. PD-L1 expression not only mediates immune evasion but also directly modulates tumor aggressiveness, as demonstrated by AKT-mTOR inhibitor studies showing reduced migration, invasion, and growth rates of DU-145 and PC-3 cells in murine models (91). The regulation of PD-1/PD-L1 in PCa extends beyond established signaling cascades, involving multifaceted crosstalk between genomic, metabolic, and immunomodulatory factors. ATM overexpression in CRPC elevates PD-L1 levels (92), while IL-6, IL-17 and TNF-α enhance PD-L1 expression via JAK/STAT3, NF-κB and AKT-dependent pathways (92, 93). The PD-L1 regulatory network integrates intrinsic factors, such as ATM mutations and PTEN loss, extrinsic stimuli like IFN-γ and epigenetic modifications, which together establish a multifaceted immunosuppressive program. This network synergizes with the prostate cancer microenvironment to promote immune escape (94).

To improve the immunosuppressive properties of “cold tumors” in PCa, researchers have conducted multiple explorations, specifically including STING agonist intervention, oncolytic virus application, and other tumor microenvironment remodeling strategies. Among these, STING (stimulator of interferon genes) serves as a key molecule in the cytoplasmic DNA sensing pathway, and its core function is to induce the production of interferons and pro-inflammatory cytokines by activating the downstream TBK1-IRF3 signaling axis, thereby initiating anti-tumor immune responses. Current research highlights several key mechanisms: (1) CDK4/6 inhibitors such as palbociclib (95) alleviate suppression of the STING–TBK1–IRF3 pathway via TBK1 or RB1 dephosphorylation; combined with STING agonists like diABZI, they markedly enhance T cell infiltration into tumors, converting immunologically “cold” tumors to “hot” ones. RNA-seq analysis of DU145 and C4–2 cells treated with palbociclib for 24 hours revealed significant activation of the cytosolic DNA-sensing pathway, cytokineingallyon receptor interactions, and Toll-like receptor signaling; in a murine RM-1 xenograft model, tumor volumes were significantly smaller following combination treatment with palbociclib and diABZI compared to monotherapies (n=5, p<0.001), with elevated infiltration of CD4+ and CD8+ T cells (n=3, p<0.001). (2) Mitochondrial DNA (mtDNA) released by senescent tumor cells (96) activates the cGAS–STING pathway in PMN-MDSCs, augmenting their immunosuppressive capacity. Specifically, tumor cells induced into senescence via OIS, PICS, or TIS release mtDNA through voltage-dependent anion channels (VDACs); this mtDNA is packaged into extracellular vesicles (EVs) and selectively internalized by PMN-MDSCs within the tumor microenvironment. Subsequent activation of the cGAS–STING pathway in PMN-MDSCs amplifies NF-κB signaling via the STING–PERK axis, ultimately enhancing immunosuppressive activity. (3) Induction of immunogenic cell death (ICD) in tumor cells via chemotherapy, radiotherapy, or physical interventions (97, 98) promotes the release of damage-associated molecular patterns (DAMPs), activating the cGAS–STING–IFN pathway and facilitating the conversion of cold tumors to hot ones. Pathological evaluation of patient samples (97) revealed substantial T cell enrichment in tumor tissues post-chemotherapy, with significantly higher densities of CD3+, CD4+, and CD8+cells in treated compared to untreated groups (P = 0.0057, 0.031, and 0.031, respectively).

Oncolytic viruses are a class of viruses that can selectively infect and kill tumor cells while stimulating the body’s specific anti-tumor immune response, and their core function lies in breaking tumor immune tolerance and remodeling the “cold” TME. A review of relevant research results shows that: the MG1-Maraba oncolytic virus (99) (carrying the STEAP vaccine) can effectively break tumor immune tolerance and realize the remodeling of the “cold” TME; reovirus (100) can target prostate cancer cells with activated Ras signaling and induce antigen-specific anti-tumor immune responses; when radiotherapy (101) is used in combination with VSV-IFNb (vesicular stomatitis virus-interferon β fusion virus), it can effectively overcome the viral resistance of tumor cells and induce long-term anti-tumor immune memory mediated by CD8+ T cells. In addition, the MG1-GFP oncolytic virus exhibits potent killing activity against human-derived prostate cancer cells (DU145, PC3, LNCaP) and mouse-derived prostate cancer cells (TRAMP-C1, TRAMP-C2); among them, TRAMP-C2 cells are highly sensitive to MG1-GFP at a low multiplicity of infection; in the study of clinical samples, 90% of patients’ biopsy samples can be effectively infected by MG1-GFP, and the yield of the virus in tumor cells is significantly higher than the initial input amount (99).

Besides the aforementioned strategies, other effective approaches for remodeling the “cold tumor” microenvironment of prostate cancer also include: inhibiting YAP1 (102) can transform the functional phenotype of cancer-associated fibroblasts, promote CD8+ T cell infiltration, and thereby enhance the efficacy of anti-PD-1 therapy; mRNA nanoparticles (103) can restore the expression of PTEN in tumor cells, induce immunogenic cell death (ICD) and systemic immune activation, while reducing the infiltration of immunosuppressive cells in the TME; targeting IRE1α (104) can achieve the remodeling of the TME, which is specifically manifested by a reduction in the number of Tregs and TAMs, an increase in the infiltration of CD8+ T cells and NK cells, and thereby exerts a synergistic effect with anti-PD-1 antibodies to effectively inhibit tumor growth.

## 4 PD-1/PD-L1 expression landscape in prostate cancer

Accumulating clinical evidence establishes that PD-1/PD-L1 expression levels independently predict treatment resistance, disease progression, and adverse prognosis in PCa. A retrospective analysis of 51 prostatectomy patients who underwent pelvic lymph node dissection revealed that PD-L1 positivity-defined as tumor cell staining in at least 1% of cells-correlated with shorter metastasis-free survival and a fourfold increased risk of distant metastasis relative to PD-L1-negative cases (105). Multivariate Cox regression analysis identified PD-L1 overexpression as an independent prognostic biomarker, associated with both elevated serum PSA levels and higher rates of surgical margin positivity (106).

PD-L1 expression patterns and clinical implications in PCa have been extensively investigated. Both primary PCa and CRPC exhibit PD-L1 expression, which correlates with poor prognostic indicators including higher Gleason scores, increased Ki-67 index, elevated PSA levels, and aggressive tumor behavior (107). Analysis of untreated prostatectomy specimens revealed PD-L1 positivity in 13.8% of localized prostate cancer cases, increasing to 26.5% in tumors with Gleason pattern 4–5; neoadjuvant therapy did not significantly alter expression levels (106). Patients with mCRPC exhibited substantially higher PD-L1 positivity (32.1%) than those with primary prostate cancer (7.7%), indicating progressive upregulation during disease progression (108). TCGA RNA sequencing data revealed complex interactions between PD-1/PD-L1 signaling, tumor purity, and immune infiltration patterns (72). Immunohistochemical studies (109) detected increased PD-L1 expression in perineural tissue, and showed an inverse relationship with CD8+ T cell density. Despite their demonstrated efficacy in melanoma, lung cancer, lymphoma, and hepatocellular carcinoma (110), PD-1/PD-L1 checkpoint inhibitors continue to show limited effectiveness in PCa. Addressing this therapeutic gap requires more comprehensive analysis of clinical trial outcomes, clarification of resistance mechanisms, and development of improved combination strategies.

This article summarizes (**Table 1**) and systematically analyzes recent research findings on the regulation of PD-1/PD-L1 expression in PCa. The analysis reveals that PD-L1 is highly expressed in enzalutamide-resistant PCa cell lines, such as C4-2B MDVR and PC-3, where it strongly correlates with enhanced immune evasion by tumor cells. Treatment with NRP2–28 *in vitro* effectively reduces PD-L1 expression and augments T cell-mediated antitumor immunity. Additionally, RelB drives PD-L1 expression via the NF-κB signaling pathway, leading to suppression of T cell immune function.

**Table 1:** PD-1/PD-L1 immunohistochemistry assay characteristics and pathologic associations in prostate cancer.

PCa Experimental Models and Biospecimens	Assay Method	Positivity Criteria	Pathologic Correlation	Ref
*In vitro* cell lines: C4-2B MDVR (Enzalutamide-resistant CRPC cells), C4-2B AbiR (Abiraterone-resistant CRPC cells), C4-2B APALR (Apalutamide-resistant CRPC cells), Myc-CaP (Androgen-sensitive PCa cells), Myc-CaP MDVR (Enzalutamide-resistant PCa cells) Murine models: Subcutaneous xenograft tumors established with Myc-CaP cells, Orthotopic prostate tumors generated using Myc-CaP MDVR cells Clinical specimens: Genomic profiling of the SU2C/PCF cohort	WB: PD-L1 protein quantification IHC: Murine tumor PD-L1 assessment (CST Ab #64988) RNA-seq/RT-qPCR: PD-L1 mRNA analysis	Quantitative FCM-IHC comparison: PD-L1+ cells proportion significantly higher in drug-resistant vs parental cells		(111)
*In vitro* cell lines: PC-3 cells (high expression of NRP2), RWPE-2 cells (to verify the association of NRP2 with PD-L1), LNCaP cells (AR-positive PCa cell line, low expression of NRP2), TRAMP-C2 cells (resistant to checkpoint inhibitors) Murine models: subcutaneous graft tumors were established using TRAMP-C2 cells. Clinical specimens: high Gleason grade PCa primaries Organoid models: neuroendocrine PCa (NEPC) metastases	IF: PD-L1/NRP2 co-localization FCM: Cell surface PD-L1 quantification RNA-seq/qPCR: PD-L1 mRNA detection	High mRNA/protein expression (RNA-seq/qPCR/FCM quantified)	Gleason score correlation: NRP2/PD-L1: Positive correlation in high-Gleason primary tumors Molecular alterations: NRP2/VEGFA/VEGFC: Gene amplification in mCRPC/NEPC Neuroendocrine features: High NRP2/PD-L1: ↑RP2/score, ↓co expression	(112)
*In vitro* cell lines: PC-3 and DU-145 (AR-negative aggressive PCa cells) Murine models: subcutaneous graft tumor and lung metastasis model using RM-1 (AR-negative) cells. Clinical specimens: PCa tissue containing 10 normal prostate tissues (control) and 46 tumor tissues with different Gleason scores (5-9).	IHC: PD-L1/RelB co-expression in PCa (CST anti-human PD-L1 Ab) WB: PD-L1 validation (Abcam anti-human/anti-mouse Ab) FCM: Tumor/T-cell PD-L1 analysis (APC/PE-conjugated PD-L1 Ab)	IHC scoring: Intensity (0-3) Proportion (0-4) Composite H-score	PD-L1 and RelB expression positively correlate with Gleason score	(113)
Clinical specimens: 239 surgical specimens of human PCa, including some metastatic lymph node specimens	IHC: PD-L1 detection (CST Ab#13684)	≥1% tumor cells with membrane/cytoplasmic staining	Gleason score association: B7-H3/HHLA2 high expression significantly correlates with Gleason ≥7 (p=0.032-0.028) TNM staging association: B7-H3/HHLA2 high expression linked to T3-T4 tumors and N+ (p=0.001-0.046)	(114)
*In vitro* cell lines: WPMY-1 cells (normal prostate cells, as a control), DU-145 and PC-3 Murine models: subcutaneous graft tumor constructed using PC-3 cells. Clinical specimens: Gleason score 6–10 PCa primary foci.	IHC: PD-L1 protein expression FACS: Cell surface PD-L1 (BioLegend Ab#329708 PE-Cy7-conjugated) qRT-PCR/WB: PD-L1 mRNA/protein validation (CST Ab#13684, GAPDH control)	≥1% on tumor cells with membrane staining	WDR5 high expression significantly correlates with Gleason 7(4 + 3)-10 WDR5 high expression linked to T3-T4 tumors and N+	(115)
*In vitro* cell lines: PC-3, DU-145 Murine models: Subcutaneous transplantation tumor was constructed using RM-1 cells. NOD/SCID humanized chimeric mouse model (transplanted human PBMCs) was constructed using PC-3 cells. Clinical specimens: 33 PCa primary and metastatic foci with Gleason score (6-10) and TNM staging (stage I-IV).	qPCR: PD-L1 mRNA levels FACS: Cell surface PD-L1 (BioLegend FITC/PE-conjugated Ab) IHC: PD-L1/pATM co-localization (Abcam Ab with pATM co-localization) WB: PD-L1/ATM/pATM detection	Fluorescence intensity/percentage-based	PD-L1 expression positively correlates with Gleason ≥8 PD-L1 overexpression observed in docetaxel-treated M1 patients	(116)
*In vitro* cell lines: BPH-1 cells (benign prostatic epithelial cells), LNCap cells (androgen-dependent prostate adenocarcinoma cells, representing early-stage tumors), C4–2 cells and CWRR1 cells (CRPC), PC-3, LASCPC-01 and NCI-H660 (neuroendocrine differentiated SCC cell lines). Murine models: Subcutaneous xenograft tumors were established using PC3 (SCC phenotype, N-cadherin positive) and LNCap (adenocarcinoma phenotype, N-cadherin negative) cells; humanized NSG mouse models were established using PC3 or LNCap cells. Clinical specimens: 30 cases of benign prostatic tissues (non-tumor areas of prostatectomy specimens), 30 cases of prostate adenocarcinoma (15 cases of low grade, Gleason score < 7; 15 cases of high grade, Gleason score ≥ 7), 18 cases of primary CRPC, and 16 cases of primary SCC.	IHC: PD-L1 expression (Rabbit anti-human Ab) FCM: Tumor surface PD-L1 (APC-conjugated Ab)	SCC group showed significantly higher PD-L1 IHC scores vs benign/adenocarcinoma/CRPC groups (threshold undefined)	PD-L1 expression restricted to neuroendocrine-differentiated SCC tissues	(117)
*In vitro* cell lines: DU-145, PC-3, 22Rv1, LNCaP, C4-2, TRAMP-C2-Ras (TRAMP-C2 mouse PCa cell line stably expressing the Ras gene) Murine models: Subcutaneous tumors were established using the TRAMP-C2-Ras cell line Clinical specimens: Analyzed 495 prostate adenocarcinoma samples (RNA sequencing data) from the TCGA database and paraffin-embedded tissues from 165 patients with primary PCa	FCM: Cell surface PD-L1 quantification WB: PD-L1 in lysates/exosomes IHC: PD-L1 expression (Ab #E1L3N/28-8)	Staining/expression significantly > control (threshold unspecified)	CD274 (PD-L1), EP300 (p300), and CREBBP gene activities inversely correlate with high Gleason scores	(72)
Clinical specimens: Cohort 1 (Only included PD-L1 positive (CPS ≥ 1)): Preferred metastatic lesion biopsy: Samples from 92% of patients (122/133) were collected within 12 months, and 80% were from metastatic lesions. Archived primary lesion specimens were allowed if metastatic lesion biopsies were not available Cohort 2 (Only included PD-L1 negative (CPS < 1)): Samples from 79% of patients (52/66) were collected within 12 months via metastatic lesion biopsy, and 92% were from metastatic lesions Cohort 3 (Regardless of PD-L1 status): Only 1 case involved a newly collected metastatic lesion biopsy (PD-L1 negative); the rest used archived primary lesion specimens (time not specified)	IHC: PD-L1 expression (PD-L1 22C3 pharmDxTM assay, Agilent Technologies)	CPS ≥1: (PD-L1+ cells/total tumor cells)×100 (includes tumor cells/lymphocytes/macrophages)		(118)
Clinical specimens: Plasma samples from patients with localized PCa (pT2/pT3 stage, non-metastatic) before surgery and 3 months after surgery	ELISA: sPD-L1/sPD-1 quantification (Thermo Fisher Scientific ELISA Kit)	sPD-L1: 7.66 pg/mL (ROC: 85% specificity, 56% sensitivity) sPD-1: 18.22 pg/mL (non-significant prognostic value)	sPD-L1: elevated in Grade Group 3 (4 + 3) vs low - grade group (23.5 vs 2.2-2.5 pg/mL, p<0.05) sPD-1: increased in pT3 (p<0.05)	(119)
*In vitro* cell lines: 22RV1 (AR-positive androgen-independent), C4-2, PC-3, DU-145, LNCaP, VCaP Murine models: Subcutaneous xenografts established using 22RV1 cells Clinical specimens: Radical prostatectomy specimens from 30 patients with PCa, including tumor tissue and adjacent normal tissue	RT-qPCR: PD-L1 mRNA quantification WB: PD-L1 immunodetection (Ab #66248,Proteintech) FCM: Cell surface PD-L1 (Ab #APC-65081,Proteintech)			(120)
Clinical specimens: Peripheral blood serum from 48 patients with mPCa and peripheral blood serum from 10 healthy males	Isolation of CTCs by Ficoll density gradient centrifugation and ISET system Triple immunofluorescence staining: Detection of PD-L protein RT-qPCR: Detection of PD-L1 mRNA			(80)
*In vitro* cell lines: RWPE-1 (normal prostate epithelial cells), LNCaP (hormone-sensitive, AR-positive), 22RV1, PC3, VCaP, DU145, RM-1 Murine models: Subcutaneous xenografts established using RM-1 cells. Clinical specimens: Paired Pca tissues and adjacent normal tissues from 95 patients.	IHC: Tumor PD-L1 localization WB: Cellular PD-L1 validation RT-qPCR: PD-L1 mRNA detection RIP-qPCR: ELAVL1-PD-L1 mRNA binding		Gleason score association: ELAVL1: positive correlation with Gleason score TNM staging: ELAVL1: positive correlation with T stage	(121)
*In vitro* cell lines: Colorectal cancer cells: RKO, HCT116 (highly expressing endogenous PD-L1), primary colorectal cancer cells; PCa cells: PC3, Du145, LNCaP; Other tumor cells: Non-small cell lung cancer A549, H1299; Triple-negative breast cancer MDA-MB-231; Human embryonic kidney cells HEK293T (for overexpression experiments) Murine models: Subcutaneous xenografts established using RM1. Clinical specimens: 81 cases of colorectal adenocarcinoma and 80 cases of adjacent normal tissues; Public databases of colon adenocarcinoma and prostate adenocarcinoma.	FCM: Cell surface PD-L1 levels WB: PD-L1 protein abundance IF: PD-L1 subcellular localization			(122)
Clinical specimens: Tumor tissues from 4 Chinese patients with primary prostate SRCC, and tumor tissues from 30 patients with conventional prostate adenocarcinoma (including 10 samples with Gleason scores of 2-4, 10 with scores of 5-7, and 10 with scores of 8-10).	IHC: PD-L1/PD-1 detection (Ab #205921/# 52587)	Staining intensity: 0 (none), 1 (weak), 2 (moderate), 3 (strong) Positivity = intensity × % positive cells		(123)
*In vitro* cell lines: EnzS1-C4-2 (C4–2 cell line, enzalutamide-sensitive) EnzR1-C4-2 (enzalutamide-resistant) Murine models: Subcutaneous xenografts established using EnzR1-C4–2 cells.	WB: PD-L1 protein expression FCM: Cell surface PD-L1 (FITC-labeled Ab)	The expression of PD-L1 is significantly higher than that of the control group (treated with EtOH)		(124)
Clinical specimens: Peripheral blood from 10 patients with mCRPC before treatment, 10 patients with mCRPC at the progressive stage after treatment, and 10 patients with mHSPC.	CellSearch System: CTC PD-L1 (CXC kit/Ventana Ab #SP142)	≥1 CTC PD-L1+ or >50% CTCs PD-L1+		(81)
*In vitro* cell lines: PC-3. Clinical specimens: PBMCs from 36 patients with PCa and 20 healthy males.	FCM: PD-1/PD-L1 in tumors/cell lines RNA-seq: PD-L1/L2 in PBMCs			(125)
Clinical specimens: 96 cases of PCa tissue and 44 cases of BPH tissue.	IHC: PD-1/PD-L1 (Ab #MAB-0654/#RMA-0732)	≥1% tumor cells/lymphocytes with moderate-strong staining or ≥10% with weak staining	PD-L1 expression: positively correlates with GS ≥8 (Tumor cells: P = 0.049, Lymphocytes: P = 0.034)	(126)
Clinical specimens: 115 cases of high-risk localized PCa surgical specimens, 3 cases of PCa biopsy specimens.	IHC: Tumor membrane PD-L1 mIF: PD-L1/CD8+ T-cell phenotype co-localization (PD-1/TCF1/TIM-3/LAG-3)	≥5% tumor membrane staining (excluding cytoplasmic)		(127)

WB, Western blotting; IHC, Immunohistochemistry; Ab, Antibody; RNA-seq, RNA sequencing; RT-qPCR, Reverse transcription quantitative real-time polymerase chain reaction; qPCR, quantitative polymerase chain reaction; Quantitative FCM-IHC, Quantitative flow cytometry-immunohistochemistry; FCM, Flow cytometry; vs, Versus; IF, Immunofluorescence; NEPC, Neuroendocrine prostate cancer; mCRPC, Metastatic castration-resistant prostate cancer; FACS, Fluorescence-Activated cell sorting; qRT-PCR, Quantitative reverse transcription polymerase chain reaction; SCC, Squamous cell carcinoma; CPS, Combined positive score; ELISA, Enzyme-linked immunosorbent assay; sPD-1/sPD-L1, Soluble PD-1/Soluble PD-L1; mPCa, metastatic PCa; CTCs, Circulating tumor cells; RIP-qPCR, RNA immunoprecipitation-qPCR; SRCC, Signet - ring cell carcinoma; mHSPC, Metastatic hormone-sensitive prostate cancer; PBMCs, Peripheral blood mononuclear cells; BPH, Benign prostatic hyperplasia.

Based on the available data, combining agents that inhibit PD-L1 expression and enhance anti-tumor immune responses—such as NRP2 or RelB inhibitors—with existing regimens may further improve therapeutic efficacy in prostate cancer. These studies, however, possess notable limitations: while each confirms the significance of PD-1/PD-L1-mediated immune escape in prostate cancer progression, they vary in their specific research emphases. Furthermore, they generally lack comparative data on drug administration sequences, making it impossible to assess how sequencing affects clinical outcomes. Existing research also omits efficacy evaluations in special populations, including elderly patients and those with hepatic impairment. Consequently, the optimal drug sequence for these populations remains to be established through subsequent studies.

## 5 Clinical exploration of PD-1/PD-L1 targeted immunotherapy for prostate cancer

Clinically, over ten PD-1 antibodies and three PD-L1 antibodies are currently approved for treating various cancers. However, PD-1/PD-L1 inhibitors exhibit limited efficacy in PCa clinical trials, likely attributable to the tumor’s “cold” immunological phenotype.

### 5.1 PD-1/PD-L1 immune checkpoint inhibitor monotherapy for prostate cancer

As core immune checkpoint inhibitors (ICIs), PD-1/PD-L1 inhibitors significantly improve clinical outcomes in diverse malignancies by disrupting tumor immune evasion. Their safety profiles and toxicity patterns, however, show substantial variability. Although these agents hold therapeutic potential for solid tumors including PCa, clinical trials report persistently low objective response rates, highlighting the urgent need to investigate resistance mechanisms and optimize treatment strategies.

#### 5.1.1 Toxicity profiles of PD-1/PD-L1 inhibitors

Overall, PD-1/PD-L1 inhibitors cause fewer adverse events (AEs) than cytotoxic chemotherapy and maintain a favorable safety profile even when AE rates increase in combination regimens (128, 129). Compared with CTLA-4 inhibitors, PD-1/PD-L1 agents are associated with lower AE frequencies; within the class, PD-1 inhibitors generally report slightly higher AE rates than PD-L1 inhibitors (130).

PD-1/PD-L1 blockade is efficacious in tumor immunotherapy but can trigger AEs because checkpoint inhibition may induce systemic immune overactivation, resulting in autoimmune-like reactions across multiple organs. The liver, gastrointestinal tract, skin, lungs, and endocrine system are most frequently affected (131, 132). The most common nonspecific AEs include fatigue, diarrhea, and pruritus (133). Across anti-PD-1, anti-PD-L1 monotherapies, and combination regimens, reported fatigue incidence ranges from 12% (134) to 71% (135); nearly 70% of patients receiving PD-1/PD-L1 inhibitors experience immune-related adverse events (irAEs), and the incidence approaches universality with combination therapy (131). Cutaneous irAEs are predominant (136, 137), most commonly presenting as lichenoid eruptions (25%) and maculopapular rash (18%).

Representative safety datasets are consistent with this profile. In a pooled analysis of 576 nivolumab-treated patients, any-grade AEs occurred in 71%, grade 3–4 events in 10%, and no treatment-related deaths were observed; most complications resolved spontaneously within weeks (138). A separate nivolumab study reported AEs in 47.3%, primarily grade 1–2 with fatal outcomes remaining exceptional (139). In contrast, the IMbassador250 trial documented AEs in 96.5% of participants (grade 3–4 in 54.3%, grade 5 in 4.3%) and serious AEs in 36.4% (140).

A comprehensive analysis of publicly available data on PD-1/PD-L1 inhibitor monotherapy in PCa demonstrates that PD-1 inhibitors (pembrolizumab, nivolumab; n = 319) (118, 139, 141) were associated with frequent adverse events including gastrointestinal toxicities (nausea, diarrhea, decreased appetite), skin and subcutaneous tissue disorders (maculopapular rash, pruritus, rash), and systemic conditions (fatigue, asthenia, weight loss). Similarly, PD-L1 inhibitors (atezolizumab, avelumab; n = 59) (142–144) commonly induced gastrointestinal toxicities (nausea, decreased appetite, colitis), dermatological manifestations (pruritus, rash, severe cutaneous reactions), and systemic symptoms (fatigue, arthralgia, infusion-related reactions). Fatigue, thyroid dysfunction (hyperthyroidism/hypothyroidism), and gastrointestinal disturbances (nausea, decreased appetite) were observed with both drug classes, with most cases being grade 1–2 in severity. While dermatological AEs (pruritus, rash) occurred frequently, ≥grade 3 reactions were generally uncommon (mostly <2%). Overall, PD-1 inhibitors exhibited higher rates of ≥grade 3 severe adverse events compared to PD-L1 inhibitors. Notably, fatal adverse events (e.g., pneumonitis, sepsis) were occasionally reported in PD-1 inhibitor studies but were not documented in PD-L1 inhibitor trials. Conversely, infusion-related reactions occurred more frequently with PD-L1 inhibitors than with PD-1 inhibitors.

According to the ASCO guideline, irAEs should be graded and managed with organ-specific attention: grade 1–2 toxicities usually do not require treatment interruption; grade 3–4 toxicities warrant permanent discontinuation and intensified immunosuppression. Myocarditis or myasthenia gravis requires escalation of care within 48 hours, whereas most endocrine toxicities rarely necessitate discontinuation (145). Multiple studies suggest that manageable irAEs correlate with better efficacy, and corticosteroid-based toxicity control does not necessarily blunt antitumor activity; upon careful reassessment, re-challenge with PD-1/PD-L1 inhibitors may be appropriate in selected cases (146, 147).

In clinical practice, a combination of baseline assessment and dynamic monitoring is recommended, routinely including complete blood count, endocrine function, and pulmonary evaluation (148). A meta-analysis across cancer types revealed that the median time to onset of irAEs ranges from 2.2 to 14.8 weeks, with severe (ever events typically occurring later; endocrine events require the longest recovery time. Follow-up frequency should be tailored based on the specific onset window and recovery pattern of each irAE (149). Throughout the treatment course, continuous patient and family education is essential, maintaining a high level of vigilance for any new symptoms (145). For PCa patients, special attention should be paid to long-term issues such as hypoandrogenism symptoms and fertility (148).

#### 5.1.2 Clinical trials of PD-1/PD-L1 inhibitor monotherapy for prostate cancer

As key ICIs, PD-1/PD-L1 inhibitors demonstrate therapeutic promise yet face clinical challenges in PCa management. The commonly used agents—nivolumab and pembrolizumab (PD-1 antibodies) and atezolizumab (PD-L1 antibody)—operate through similar mechanisms but exhibit distinct response profiles and efficacy patterns in PCa treatment.

Nivolumab (150, 151) demonstrates high PD-1 binding affinity on T cells, inducing their activation and subsequent tumor cell apoptosis through T cell-mediated cytotoxicity. In a single-arm, multicenter phase II trial (139) involving 38 mCRPC patients, nivolumab treatment yielded a PSA50 response rate (≥50% PSA decline) of merely 10.5% (4 patients), with an overall objective response rate of 26%. A phase I study (152) of nivolumab in 17 mCRPC patients reported no objective responses.

Pembrolizumab (153), a humanized anti-PD-1 monoclonal antibody, augments T cell-mediated antitumor immunity through PD-1/PD-L1 axis blockade. This immunotherapeutic agent demonstrates efficacy across multiple malignancies, including melanoma (154), non-small cell lung cancer (155), and lymphoma (156). Graff (157) conducted a phase II trial evaluating pembrolizumab in mCRPC, observing PSA reductions below 0.1 ng/ml in 3 of 10 treated patients. The KEYNOTE-028 trial (141) enrolled PD-L1-positive patients, with pembrolizumab treatment yielding stable disease in 8 of 23 participants and median PFS and OS durations of 3.5 and 7.9 months, respectively.

Atezolizumab, a humanized IgG1 monoclonal antibody, prevents T cell exhaustion through PD-L1 blockade mediated by antibody-dependent cellular cytotoxicity (158). In a phase Ia trial of 35 mCRPC patients (142), RECIST 1.1 criteria identified one partial responder, while a second patient met the immune-related response criteria. This review synthesizes published clinical trial data on PD-1/PD-L1 inhibitor monotherapy for prostate cancer (**Table 2**).

**Table 2:** Therapeutic potential of PD-1/PD-L1 inhibitors for prostate cancer.

Target	Checkpoint Inhibitors	Phase	Number of Patients	Primary Endpoint	Results	Trial/NCT
PD-1	Nivolumab	II	38	PSA50 response rate	PSA50 response rate: 10.5% (4/38)	NCT 03040791 (139)
PD-1	Pembrolizumab	II	258	ORR	ORR: 5% (95% CI: 2%–11%, Cohort 1) vs 3% (95% CI: 1%–11%, Cohort 2)	KEYNOTE-199(NCT02787005) (118)
PD-1	Pembrolizumab	II	86	ORR	ORR: 53% (46/86, 95%CI: 42%–64%) CR: 21% (18/86) PR: 33% (28/86) DCR: 77% (66/86, 95%CI: 66%–85%)	NCT01876511 (159)
PD-1	Pembrolizumab	Ib	23	PFS, OS	Median PFS: 3.5 mo (95%CI: 1.7–6.5) 6-mo OS rate: 73.4% 12-mo OS rate: 36.7%	NCT02054806 (141)
PD-1	Atezolizumab	II	35	safety	Any-grade TRAEs: 60.0% (21/35) Grade ≥3 TRAEs: 11.4% (4/35)	NCT01375842 (142)
PD-L1	Avelumab	I	18	BOR	No CR or PR cases Among 17 evaluable patients: SD: 70.6% PD: 29.4% SD duration >24 weeks: 41.2%	NCT01772004 (143)
PD-L1	Avelumab	II	8	PSA50 response rate	No patients achieved PSA50 response	NCT03770455 (144)

PSA50, prostate-specific antigen 50% decrease from baseline; ORR, objective response rate; PR, partial responses; CR, complete responses; DCR, disease control rate; PFS, progression-free survival; TRAEs, treatment-related adverse events; BOR, best overall response; PD, progressive disease; SD, stable disease; mo, month.

### 5.2 Combination therapies involving PD-1/PD-L1 inhibitors for prostate cancer

As mentioned above, PD-1/PD-L1 inhibitors demonstrate clinical efficacy against multiple tumor types, but their effectiveness in PCa remains limited. Growing insights into immunotherapy’s regulatory mechanisms have driven increasing interest in combination treatment strategies. Current approaches integrate immunotherapy with ADT, radiotherapy, chemotherapy, or other immunotherapeutic agents (**Table 3**, for more detailed information, please refer to the **Supplementary Material**).

**Table 3:** Combined therapeutic approaches involving PD-1/PD-L1 inhibitors and other modalities for prostate cancer.

Combination Agents	Phase	Efficacy		Adverse rate	Trial/NCT
Pembrolizumabb + Enzalutamide	II	PSA reduction ≥50%: 18% (5/28) OR: 25% (3/28) Median PSA-PFS: 3.8mo (95% CI: 2.8mo–9.9mo Median OS: 21.9mo (95% CI: 14.7mo–28.4mo). Extended median OS of ‌41.7 mo‌ (95% CI:22.16–NR )among responders		Grade 2–3 irAEs: 25% (7/28) Other Grade 3–4 AEs: 25%(7/28)	NCT 02312557 (79)
Pembrolizumab + ADXS31-142	I/II	ORR: 0% Median PFS: 2.2 mo (95% CI: 0.8–7.4) with ADXS31-142 monotherapy vs. 5.4 mo (95% CI: 2.3–7.9) with combination therapy Median OS:‌ 7.8 mo (95% CI: 4.4–18.5) with ADXS31-142 monotherapy vs. 33.7 mo (95% CI: 15.4–NE) with combination therapy		TRAEs:‌ All patients (n=50) experienced ≥1 TRAE, predominantly grade 1–2 Grade ≥3 AEs:‌ 38.5% with ADXS31-142 monotherapy vs. 29.7% with combination therapy	NCT 02325557 (160)
Pembrolizumab + Enzalutamide	Ib/II	PSA response rate: 24% (95% CI: 16–33) with 26% (95% CI: 13–43) in patients with measurable disease ORR: 11% (95% CI: 2.9–25), comprising 2 CR and 2 PR Median rPFS: 6.0 mo (95% CI: 4.1–6.3) Median OS :20 mo (95% CI: 17–24)		All-grade TRAEs: 92% (94/102) Grade ≥3 TRAEs: 43% (44/102) irAEs: 37% (38/102), with grade ≥3 events in 22% (22/102; primarily severe cutaneous reactions) Fatal TRAEs: 1 case (1/102, 1.0%)	KEYNOTE-365(NCT02861573) (129)
Pembrolizumab + Docetaxel	III	rPFS: 8.6 mo (95% CI: 8.3–10.2) with EXP vs. 8.3 mo (95% CI: 8.2–8.5) with CTRL OS: 19.6 mo (95% CI: 18.2–20.9) with EXP vs. 19.0 mo (95% CI: 17.9–20.9) with CTRL TFST: 10.7 mo with EXP vs. 10.4 mo with CTRL PSA response rate: 44.5% (95% CI: 40.0–49.1) with EXP vs. 45.7% (95% CI: 41.2–50.2) with CTRL ORR: 33.5% (95% CI: 27.0–40.4) with EXP vs. 35.3% (95% CI: 29.0–42.0) with CTRL PSA-PFS: 6.9 mo with EXP vs. 7.0 mo with CTRL DOR: 6.3 mo (range: 3.4–21.2) with EXP vs. 6.2 mo (range: 2.0–13.1) with CTRL		Grade ≥3 TRAEs: 43.2% (EXP) vs. 36.6% (CTRL) irAEs: 19.1% (EXP) vs. 10.5% (CTRL)	KEYNOTE-921(NCT03834506) (161)
Pembrolizumab + Docetaxel + Prednisone	Ib/II	PSA response rate: 34% ORR: 23% (12/52; 95% CI: 13–37) DCR: 54% (95% CI: 44–64) Median rPFS: 8.5 mo (95% CI: 8.3–10) Median OS : 20.2 mo (95% CI: 17–24)		All-grade TRAEs: 96% (100/104) Grade ≥3 TRAEs: 44% (46/104) irAEs: 33% (34/104), with grade ≥3 irAEs in 8.7% (9/104) Treatment-related deaths: 2 cases (1.9%, both immune-mediated pneumonitis)	KEYNOTE-365(NCT02861573) (162)
Pembrolizumab + Olaparib	III	rPFS: 4.4 mo (95% CI: 4.2–6.0) with EXP vs. 4.2 mo (95% CI: 4.0–6.1) with CTRL OS: 15.8 mo (95% CI: 14.6–17.0) with EXP vs. 14.6 mo (95% CI: 12.6–17.3) with CTRL TFST: 7.2 mo (95% CI: 6.7–8.10) with EXP vs. 5.7 mo (95% CI: 5.0–7.1) with CTRL ORR: 16.8% (95% CI: 12.3–22.1) with EXP vs. 5.9% (95% CI: 2.4–11.7) with CTRL PSA50 response rate: 16.6% (95% CI:13.4–20.1) with EXP vs. 19.0% (95% CI: 14.3–24.5) with CTRL		Grade ≥3 TRAEs: 34.6% (EXP) vs 9.0% (CTRL) TRAEs: 10.8% (EXP) vs 1.6% (CTRL) Fatal TRAEs: 4 cases, including hepatitis and pneumonia (EXP) vs 0(CTRL) Any-grade irAEs: 18.1% (EXP) vs 5.5% (CTRL) Grade ≥3 irAEs: 5.1% (EXP) vs 1.2% (CTRL)	KEYLYNK-010(NCT03834519) (163)
Pembrolizumab + Olaparib	Ib/II	PSA response rate: 15% (15/102) ORR: 8.5% (5/59; 95% CI: 2.8–19) DCR: 26% (95% CI: 18–36) Median rPFS: 4.5 mo (95% CI: 4.0–6.5) Median OS: 14 mo (95% CI: 10.4–18.2)		All-grade TRAEs: 91% (93/102) Grade ≥3 TRAEs: 48% (49/102) irAEs: 12% (12/102), including grade 3–5 pneumonitis (n=2) Treatment-related deaths: 2 cases (1.9%; myocardial infarction and unknown etiology)	KEYNOTE-365(NCT02861573) (164)
Pembrolizumab + Anti-CD3 * Anti-HER2 Bispecific Antibody-Armed T Cells	II	Median PFS: 5 mo (95% CI: 4–NR) Median OS: 31.6 mo (95% CI: 7.4–NR) PSA decline ≥25%: 43% (6/14)		Grade 1–2 infusion-related reactions: 100% (14/14) Grade 3 AE: fatigue (n=2), altered mental status (n=1)	NCT03406858 (165)
Pembrolizumab + Radium-223	II	TIL density: CD4⁺ cells/HPF: -0.7 (-9.3 to 4.7, Combination group) vs -0.1 (-11.1 to 3.7,Monotherapy group) CD8⁺ cells/HPF: -0.6 (-7.4 to 5.3, Combination group) vs -1.3 (-3.1 to 4.8,Monotherapy group) Median rPFS: 6․1 mo (95% CI: 2․7–11․0,Combination group) vs 5․7 (95% CI: 2․6–NR,Monotherapy group) Median OS: 16.9 mo (95% CI: 12.7–NR,Combination group) vs 16.0 (95% CI: 9.0–NR,Monotherapy group)		Grade ≥3 TRAEs: 17.0%(5 cases, Combination group) vs 8.3%(1 case, Monotherapy group) Pembrolizumab-related grade ≥3 AEs: Pneumonitis (n=2, grade 2-4); Diarrhea (n=1, grade 3); Aspartate aminotransferase elevation (n=1, grade 4)	NCT03093428 (166)
Pembrolizumab + 177Lu-PSMA-617	I	Cohort B( n=25) : ORR: 56% (14/25; 95% CI: 35–76) PSA50 response rate: 44% (19/43; entire cohort; 95% CI: 29–59) Median PFS: 6.9 mo (95% CI: 3.9–7.0) Median DOR: 8.1 mo (range: 6.0–10.0)		Grade ≥3 TRAEs: 5% (2/43) One case of fatal inhalation pneumonia (non-treatment-related)	NCT03805594 (167)
Pembrolizumab + BXCL701	IIa	CR: 33.3%(5/15 patients, including 4 cases of RECIST-defined PR and 1 case of PSA50 or CTC conversion)		No unexpected irAEs	NCT03910660 (168)
Pembrolizumab + MVI-816	II	6-mo rPFS: 44.4%(arm3) vs 61.5%(arm4) Median rPFS: 5.6 mo (arm3) vs 8.1 mo (arm4). Overall: 5.6 mo (95% CI: 5.4–10.8,n=40) PSA50 response rate: 10% (4/40, Overall) PR: 1 patient(arm3) vs 0 patient(arm4) OS: 22.9 mo (95% CI: 16.2–25.6, Overall)		Any-grade irAEs: 48% (arm3) vs 48% (arm4) Grade ≥3 TRAEs: 4 cases (arm3) vs 13 cases (arm4)	NCT02499835 (169)
Pembrolizumab + Cisplatin-based chemotherapy	Ib	cohort 2 ORR: 43% (95% CI: 6.0–80.0) 12-mo PFS: 43% ( 95% CI: 18-100) 12-mo OS: 71% (95% CI: 45-100)		Grade ≥ 3 TRAEs: 40%(6/15) SAEs: 5 cases (fatigue, pyelonephritis, epilepsy, etc.) No treatment-related deaths or discontinuations due to toxicity	NCT03582475 (170)
Pembrolizumab + GSK2636771	I/II	PR: 18.2% (2/11,tumor reductions of 56% and 59%, respectively) SD: 1 patient, tumor reduction of 18%, ongoing treatment for 15.8 months PFS: 2 PR patients, PFS >12 mo, durations of 24.1 mo and 13.6 mo, respectively PSA50 response rate: 2 PR patients		TRAEs: Grade 1–2: Diarrhea (33%), Rash (42%) Grade 3: Rash (4 cases, 2 of which were immune-related bullous pemphigoid), Hyperphosphatemia (1 case)	NCT01458067 (171)
Nivolumab + Ipilimumab	II	PSA50 Response Rate: 9% (95% CI: 1–28%, 2/23, Cohort A) vs 0% (0/14,Cohort C) Median PSA-PFS: 7.0 mo (95% CI: 3.6–11.4, Cohort A) vs 4.5 mo (95% CI: 3.4–13.8, Cohort C) Median OS: 9.0 mo (95% CI: 6.2–12.3, Cohort A) vs 13.8 mo (95% CI: 3.6–NR, Cohort C) ORR: No objective responses observed in either cohort		Any-Grade AEs: 81.3% (26/32, Cohort A) vs 60% (9/15, Cohort C) Grade ≥3 AEs: 9.4% (3/32, Cohort A) vs 0% (0, Cohort C) SAEs: 9.4% (3/32, Cohort A) vs 0% (0, Cohort C)	IMPACT(NCT03570619) (172)
Nivolumab + Ipilimumab	II	ORR: 25%(95% CI: 11.5–43.4, 8/32, Cohort 1) vs 10% (95% CI: 2.1–26.5,3/30, Cohort 2) Median rPFS: 5.5 mo (95% CI: 3.5–7.1, Cohort 1) vs 3.8 mo (95% CI: 2.1–5.1, Cohort 2) Median OS: 19.0 mo (95% CI: 11.5–NE,Cohort 1) vs 15.2 mo (95% CI: 8.4–NE, Cohort 2)		Any-Grade TRAEs: 93.3% (42/45,Cohort 1) vs 95.6% (43/45,Cohort 2) Grade ≥3 TRAEs: 42.2% (19/45,Cohort 1) vs 53.3% (24/45,Cohort 2) Treatment-Related Deaths: 2 cases (myocarditis, sudden death, Cohort 1) vs 2 cases (sepsis, interstitial lung disease, Cohort 2)	CheckMate 650(NCT02985957) (173)
Nivolumab + Ipilimumab	II	DCR >6 mo: 38% (95% CI: 27–51) ORR: 38% (95% CI: 22–55), all PR PSA50 response: 47% (95% CI: 34–60) Median PFS: 4.0 mo (95% CI: 3.5–12.0)		Any-Grade TRAEs: 97% (67/69) Grade ≥3 TRAEs: 48% (33/69) Grade 4 TRAEs: 1 case (fatal gastrointestinal perforation), 1 case chose euthanasia due to grade 4 toxicity TRAEs: 20% (14/69)	INSPIRE(NCT04717154) (174)
Nivolumab + pTVGHP	II	PSA response rate: 0% (95% CI: 0–17%, 0/19) PSA50 response rate: 21% (95% CI: 8.6–43%,4/19) Median PSA doubling time: 5.9 mo (Pre-treatment) vs 25.6 mo (On-treatment) vs 9.0 mo (Post-treatment) 2-year MFS: 79% (95% CI: 57–91%,15/19)	Grade ≥3 AEs: 26%(5/19) irAEs: 47%(9/19)		NCT03600350 (175)
Nivolumab + Docetaxel	II	ORR: 40.0% (95% CI: 25.7-55.7) PSA50 response rate: 46.9% (95% CI: 35.7-58.3) Median rPFS: 9.0 mo (95% CI: 8.0-11.6) Median OS: 18.2 mo (95% CI: 14.6-20.7)	Any-grade TRAEs: 95.2% (80/84) Grade 3–4 TRAEs: 47.6% (40/84) Treatment-related deaths: 3.6% (3/84); 1 case of pneumonitis (attributed to nivolumab), 2 cases of pneumonia (attributed to docetaxel)		CheckMate 9KD(NCT03338790) (128)
Nivolumab + Rucaparib	II	ORR: 10.3% (95% CI: 3.9–21.2,Cohort A1) vs 15.4% (95% CI: 5.9–30.5%, Cohort A2) PSA50 response rate: 11.9%(95% CI:5.9–20.8,Cohort A1) vs 27.3% (95% CI: 17.0–39.6, Cohort A2) Median rPFS: 4.9 mo (95% CI: 3.7–5.7,Cohort A1) vs 8.1 mo (95% CI: 5.6–10.9,Cohort A2) Median OS: 13.9 mo (95% CI: 10.4–15.8,Cohort A1) vs 20.2 mo (95% CI: 14.1–22.8,Cohort A2)	Any-grade TRAEs: 93.2% (Cohort A1) vs 90.1% (Cohort A2) Grade 3–4 TRAEs: 54.5% (Cohort A1) vs 50.7% (Cohort A2)		CheckMate 9KD (NCT03338790) (176)
Nivolumab + Ipilimumab ± Enzalutamide	II	PSA50 response rate: 13.3%(2/15, 95%CI: 2.5-39,Cohort 1 ) vs 0.0%(0/15, 95%CI: 0.0-23.9,Cohort 2) vs 6.7%(2/30, 95%CI: 0.8–22.4, Combined) Median PSA-PFS: 3.0 mo (95%CI: 2.1–NR, Cohort 1) vs 2.7 mo (95%CI: 2.1–5.9, Cohort 2) vs 2.8 mo (95%CI: 2.1–4.1, Combined) Median rPFS: 3.7 mo (95%CI: 2.8–7.5, Cohort 1) vs 2.9 mo (95%CI: 1.3–5.8, Cohort 2) vs 3.0 mo (95%CI: 2.7–5.5, Combined) ORR: 25.0% (2/8, 95%CI: 6.3–59.9%, Cohort 1) vs 0.0% (0/9, 95%CI: 0.0–34.5%, Cohort 2) vs 11.8% (2/17, 95%CI: 2.0–35.6%, Combined) Median OS: 8.2 mo (95%CI: 5.5–10.4, Cohort 1) vs 14.2 mo (95%CI: 8.5–NA, Cohort 2) vs 9.5 mo (95% CI: 8.1–11.4, Combined)	Grade 3–4 AEs: 46% (7/15, Cohort 1) vs 53% (8/15, Cohort 2) irAEs: 33% (5/15, Cohort 1) vs 47% (7/15, Cohort 2)		NCT02601014 (52)
Atezolizumab + Cabozantinib	Ib	ORR: 23.5% (95%CI: 17.0–32.0%) Median DOR: 8.3 mo (95%CI: 4.6–11.0) Median PFS: 5.5 mo (95%CI: 4.3–6.6) Median OS: 18.4 mo (95%CI: 14.3–24.7)	Any-grade AEs: 95% (126/132) Grade ≥3 AEs: 55% (72/132) SAEs: 56% (74/132) TRAEs: 21% (28/132); cabozantinib alone: 18%, atezolizumab alone: 14% Treatment-related deaths: 2.3% (3/132); 1 case of dehydration attributed to treatment		COSMIC-021(NCT03170960) (177)
Atezolizumab + Sipuleucel-T	Ib	ORR: 4.3% (1/23; 1 PR observed in Arm 2) DCR: 21.7% (5/23; 3 SD in Arm 1, 1 PR and 1 SD in Arm 2) Median rPFS: 3.3 mo (95% CI: 2.7–7.8, Arm 1) vs 2.9 mo (95% CI: 2.6–5.5, Arm 2) vs 3.0 mo (95% CI: 2.8–5.6, Overall) Median OS: NR (95% CI: 11.1–NR, Arm 1) vs 21.4 mo (95% CI: 16.6–NR, Arm 2)	TRAEs: 83.8% (31/37) Grade ≥3 TRAEs: 18.9% (7/37) irAEs: 13.5% (5/37, all Grade 1–2)		NCT03024216 (178)
Atezolizumab + Enzalutamide	III	Median OS: 15.2 mo (95% CI: 14.0–17.0, Combo arm) vs 16.6 mo (95% CI: 14.7–18.4, CTRL) Median rPFS: 4.2 mo (95% CI: 4.1–5.3, Combo arm) vs 4.1 mo (95% CI: 3.7–4.5, CTRL) Median PSA progression time: 2.8 mo (95% CI: 2.8–2.9, Combo arm ) vs 2.8 mo (95% CI: 2.8–2.9, CTRL) ORR: 13.7% (95% CI: 8.4–20.7, Combo arm ) vs 7.4% (95% CI: 3.7–13.0, CTRL)	Any-grade AEs: 96.5%(Combo arm) vs 91.8%(CTRL) Grade 3–4 AEs: 54.3%(Combo arm) vs 34.8%(CTRL) irAEs: 0.8% (Combo arm) vs 0%(CTRL)		IMbassador250(NCT 03016312) (140)
Atezolizumab + Radium-223	Ib	ORR: 6.8% (95% CI: 1.4–18.7, Overall) Median rPFS: 3.0 mo (95% CI: 2.8–4.6, Overall) PSA50 Response Rate: 4.5% (95% CI: 0.6–15.5, Overall) Median OS: 16.3 mo (95% CI: 10.9–22.3, Overall)	Any-grade AEs: 100% (44/44) Grade 3–4 AEs: 52.3% (23/44) irAEs: 18.2% (8/44) Treatment-related deaths: 6.8% (3/44, all attributed to atezolizumab; no additional risk observed with radium-223)		NCT02814669 (179)
Avelumab + Stereotactic ablative body radiotherapy	II	DCR: 48% ( 15/31, (95% CI:30–67) ORR: 31%( 5/16, 95% CI: 11–59) Median rPFS: 8.4 mo (95% CI: 4.5–NR, Overall) Median OS: 14.1 mo (95% CI: 8.9–NR, Overall)	Any-grade TRAEs: 90% (28/31) Grade ≥3 TRAEs: 16% (5/31)		ANZCTR number(ACTRN12618000954224) (180)
Avelumab + Carboplatin	Ib	PSA50 Response Rate: 7.7% (2/26) ORR: 17.6% (3/17) Median rPFS: 6.6 mo (95% CI: 4.28–9.01) Median OS: 10.6 mo (95% CI: 6.68–NR)	Grade 3–4 TRAEs: 73% (19/26) SAEs: 7.7% (2/26) Treatment discontinuation: 3.8% (1/26, due to Grade 4 thrombocytopenia)		EudraCT number(2017-004552-39) (181)
Nivolumab + BAT	II	PSA50 Response Rate: 40% (18/45, 95% CI: 25.7–55.7) ORR: 24% (10/42) Median rPFS: 5.6 mo (95%CI: 5.4–6.8) Median OS: 24.4 mo (95%CI: 17.6–31.1)	Grade 3 TRAEs: 11% (5/45)		COMBAT(NCT03554317) (182)

rPFS, radiographic progression-free survival; mo, month; OR, objective response; OS, Overall survival; PSA-PFS, PSA progression-free survival; AEs, adverse events; irAEs, immune-related adverse events; ORR, objective response rate; CR, complete responses; PR, partial responses; EXP, experimental arm; CTRL, control arm; Combo arm, Combination arm; DCR, disease control rate; TFST, treatment-free survival time; NR, not reached; SD, stable disease.

Although ADT, radiotherapy, and chemotherapy are all conventional treatment modalities for prostate cancer, some patients still experience disease progression during treatment. Current research data indicate that the occurrence of ADT resistance (27, 183–186) is significantly associated with abnormalities in the AR pathway (including low AR activity, upregulation of AR-V7, and inhibition of AR target genes caused by CHD1 deletion), abnormal regulation of myeloid cells (elevated neutrophil-to-lymphocyte ratio (NLR), enrichment of CXCL1/2/8, and infiltration of polymorphonuclear myeloid-derived suppressor cells (PMN-MDSCs)), and activation of the protein stabilization axis (KIF15-USP14-AR/AR-V7). Notably, reduced AR pathway activity is a necessary condition for resistance to AR signaling inhibitors (186); in addition, PMN-MDSCs can promote resistance to AR signaling inhibitors by activating AR-V7 (27), while KIF15 can enhance the resistance of tumor cells to enzalutamide by stabilizing the protein levels of AR/AR-V7 (184).

The mechanism of radiotherapy resistance (187–190) is closely associated with the high expression of metabolic markers (GLS1, LDHA), upregulation of stem cell-related markers (ALDH1A1), activation of DNA repair genes (PLK3), and high-risk status of CAF-related gene signatures (BCRFS/MFS signatures). Specifically, GLS1 inhibitors can significantly enhance the radiosensitivity of PCa cells (187); high expression of ALDH1A1 is closely associated with bone metastasis of PCa and post-radiotherapy recurrence risk (190); meanwhile, CAF-related gene signatures have high predictive efficacy for post-radiotherapy biochemical recurrence and distant metastasis (188).

Chemotherapy resistance (115, 191, 192) (with docetaxel and cisplatin resistance as the main research objects) occurs in association with intestinal microbiota dysbiosis (enrichment of Proteobacteria), activation of the inflammatory signaling axis (LPS-NF-κB-IL6-STAT3), abnormalities of the metabolic regulatory axis (circARHGAP29-IGF2BP2/c-Myc-LDHA), and dysregulation of the epigenetic regulator (WDR5). Among these, STAT3 inhibitors can increase the apoptosis rate of docetaxel-resistant cells by 2–3 folds (191); silencing circARHGAP29 can effectively reverse the resistance of tumor cells to docetaxel (192); while WDR5 inhibitors can enhance the sensitivity of cells to cisplatin by downregulating the expression of the DNA repair gene (XRCC2) (115).

The mechanisms of immunotherapy resistance (28, 193–196) involve tumor suppressor gene deletion (e.g., PTEN deletion leading to a “cold tumor” phenotype in tumors), abnormal transcription factor regulation (FOXA1 inhibiting the IFN signaling pathway), activation of chromatin effectors (Pygo2-Kit-Ido1 axis), and myeloid cell abnormalities (enrichment of SPP1hi-TAMs). Regarding specific mechanisms, SPP1hi-TAMs can inhibit the anti-tumor function of CD8+ T cells through the adenosine-A2AR pathway (28); while Pygo2 inhibitors can reverse immune checkpoint inhibitor resistance by enhancing the infiltration of cytotoxic T lymphocytes in tumor tissues (195).

#### 5.2.1 PD-1/PD-L1 inhibitor therapy combined with ADT for prostate cancer

Androgen receptor (AR) signaling plays a critical role in driving PCa initiation and progression, thereby establishing AR signaling as a principal therapeutic target. This pathway modulates T-cell function through PTPN1 transcriptional activation while suppressing Th1 cell activity via JAK/STAT pathway downregulation (100). As the cornerstone treatment for metastatic PCa, ADT remodels tumor immune infiltration (197), potentially enhancing PD-1/PD-L1 inhibitor sensitivity (198).

The KEYNOTE-199 trial (199) demonstrated that pembrolizumab combined with enzalutamide in mCRPC yielded a 22% PSA response rate, a 51.1% disease control rate for bone-only metastases, and a median OS of 20.8 months. Phase II studies (199) and the KEYNOTE-365 trial (129) later corroborated the antitumor activity of this regimen. However, the IMbassador250 trial (140) found no significant benefit in OS, PFS, or PSA progression time from adding atezolizumab to enzalutamide, except among patients with high CD8 T-cell infiltration and PD-L1 expression, who showed improved progression-free survival. Investigators have also explored alternative strategies to conventional ADT combinations, such as bipolar androgen therapy. In the COMBAT trial, nivolumab paired with cyclic testosterone modulation achieved a 40% PSA50 response rate (18/45), with median OS reaching 24.4 months and radiographic PFS (rPFS) lasting 5.6 months (182).

#### 5.2.2 PD-1/PD-L1 inhibitor therapy combined with radiotherapy for prostate cancer

Radiotherapy remains a cornerstone treatment for diverse tumor types. By damaging tumor cell DNA and inducing cell death, it modifies the TME while increasing immunogenicity through enhanced release of tumor-associated antigens and upregulation of tumor suppressor proteins and cytokines (200–202). These effects collectively stimulate both adaptive and innate immune responses. Current radiotherapy approaches primarily comprise external beam radiotherapy and targeted radionuclide therapy, distinguished by their dosimetric characteristics.

Radium-223 (203), an alpha-emitting radionuclide, induces DNA double-strand breaks and targets tumor-associated osteoblasts by mimicking calcium-phosphorus complexes, ultimately altering the PCa microenvironment. In mCRPC patients receiving pembrolizumab combination therapy (166), median OS and rPFS reached 16.9 and 6.1 months, respectively, versus 16.0 and 5.7 months with radiotherapy alone. The immune cell infiltration profiles did not differ significantly between treatment groups.

^177^Lu-PSMA-617 combines the beta-emitting radionuclide Lutetium-177 with PSMA-617, a high-affinity ligand targeting prostate-specific membrane antigen (PSMA) (204),. In mCRPC patients, Aggarwal et al. (167) reported improved therapeutic outcomes when this agent was paired with pembrolizumab. Administering pembrolizumab 28 days after ^177^Lu-PSMA-617 treatment produced optimal results, with 56% (14/25) of patients showing objective responses (>50% PSA reduction), a median PFS of 6.9 months, and an OS extending to 28.2 months. Another study examined the efficacy of stereotactic ablative radiotherapy (SABR) combined with avelumab (a PD-L1 inhibitor) (180).

#### 5.2.3 PD-1/PD-L1 inhibitor therapy combined with chemotherapy for prostate cancer

Chemotherapy remains a cornerstone of tumor treatment, primarily through tumor cell destruction that releases antigens and damage-associated molecular patterns. This process activates dendritic cells, enhancing antigen cross-presentation and triggering specific immune responses (205). Concurrently, chemotherapy diminishes immunosuppressive cell populations, including MDSCs and Tregs (206, 207). It also stimulates CXCL10 production within tumor tissues, a chemokine that recruits T cells and potentiates tumor-infiltrating lymphocyte activity through its dual chemotactic and immunomodulatory functions (208). As a steroidal antimitotic agent, docetaxel exerts its apoptotic effects by binding β-tubulin and disrupting microtubule dynamics during cell division (97).

In the CheckMate 9KD trial, nivolumab plus docetaxel was administered to patients with mCRPC (128). The combination achieved an ORR of 40.0% among 84 treated patients, with a median time to response of 2.0 months. The median response duration was 7.0 months, and median rPFS stood at 9.0 months. Median OS reached 18.2 months.

The KEYNOTE-921 trial investigated pembrolizumab plus docetaxel (161, 209) in mCRPC patients. Of the 1030 randomized participants, 515 were treated with the pembrolizumab-docetaxel combination. The ORR was 33.5%, with a median response duration of 6.3 months. Median rPFS survival reached 8.6 months, while the OS was 19.6 months. Previous studies have also examined pembrolizumab combined with platinum-based chemotherapy (170) for small cell or neuroendocrine PCa.

#### 5.2.4 Other combinatorial immunotherapy approaches for prostate cancer

The limited efficacy of monotherapy with immune checkpoint inhibitors has driven research toward combination approaches. CTLA-4, primarily expressed on activated T cells and regulatory T cells, suppresses T cell activity through mechanisms such as PI3K-Akt pathway inhibition and CD80/CD86 degradation (210). In the CheckMate 650 trial (173), nivolumab combined with ipilimumab achieved a 10.0% objective response rate, 6.7% complete response rate, and 13.3% disease control rate, with a median rPFS of 3.8 months in mCRPC patients previously treated with chemotherapy.

Comparative analysis of different combination regimens reveals that for patients with mCRPC progressing after enzalutamide, the sequence of pembrolizumab combined with enzalutamide yields a significantly higher PSA response rate than other combinations; its mechanism-checkpoint blockade plus ADT-may enhance antitumor immune activity and improve overall efficacy. However, among mCRPC patients who have received no more than two prior lines of metastatic therapy, this combination should be avoided due to both reduced efficacy and increased side effects, including a higher incidence of grade 2–3 immune-related adverse events.

To improve the clinical outcomes of immunotherapy while minimizing toxicity, researchers are investigating novel combination therapies, including pembrolizumab with docetaxel and prednisone (162) as well as nivolumab and ipilimumab paired with enzalutamide (52). Although ORR and survival times show improvement, treatment-related AEs remain a growing concern. These findings offer robust clinical evidence to advance precision immunotherapy for PCa and inform future personalized combination approaches.

## 6 Current challenges and future perspectives

The development of ICIs targeting the PD-1/PD-L1 axis has expanded therapeutic options for advanced PCa, although clinical outcomes continue to demonstrate limited efficacy. As a “cold tumor,” PCa demonstrates robust immune resistance, resulting in significantly poorer responses to PD-1/PD-L1 inhibitors compared to those observed in lung cancer (211), melanoma (212), squamous cell carcinoma (213), or hepatobiliary and colorectal malignancies (214). To enhance ICI efficacy, overcoming this resistance is imperative. Current strategies integrate ICIs with conventional treatments like ADT, radiotherapy, or chemotherapy, as well as emerging modalities such as alternative checkpoint inhibitors, cancer vaccines, and targeted therapies. While these combinations can amplify antitumor immunity, they frequently exacerbate treatment-related toxicities, underscoring the need for more refined patient selection. Major challenges involve managing overlapping adverse effects, establishing optimal dosing sequences, and mitigating irAEs arising from dual checkpoint blockade. Future studies should address these constraints through rigorously designed clinical trials.

First, PCa immune evasion arises from a complex regulatory network spanning multiple pathways. The JAK–STAT and PI3K–AKT–mTOR cascades directly modulate PD-L1 expression, while metabolic reprogramming and epigenetic modifications synergistically promote immune escape (215, 216). These insights inform the development of targeted immunotherapies. However, the substantial interpatient and intratumoral heterogeneity of PCa limits one-size-fits-all approaches. Tumor heterogeneity further compounds complexity in an already heterogeneous patient population (217). A single biomarker (e.g., PSA, an individual gene mutation, or a solitary imaging feature) is insufficient to predict responses to combination therapies; integrating multi-omics data—including genomics, transcriptomics, proteomics, radiomics, and clinical information—enables a more comprehensive evaluation of patient responses, improves predictive accuracy, and supports effective screening for immunotherapy-based combination regimens (218–220). For example, a predictive model based on macrophage-related marker genes (221) has demonstrated potential in guiding the precise selection of combination therapies, enabling the prediction of responses to ICIs, identifying chemotherapy-sensitive subgroups, and assisting in the combined application of targeted drugs. Future studies should prioritize predictive model development through integrated multi-omics analyses of genomic, transcriptomic, and proteomic profiles.

Second, clinical consensus is lacking on optimal timing and sequencing of immunotherapy. Emerging evidence suggests that initiating ICIs during the metastatic hormone-sensitive PCa (mHSPC) phase may improve outcomes, but confirmation is needed. For advanced disease, clinicians must carefully integrate immunotherapy with standard modalities (e.g., ADT, taxane-based chemotherapy, radiotherapy) (178). Robust response assessment systems and dynamic monitoring remain priorities, including identification of precise biomarkers (174, 222) such as CDK12 biallelic inactivation (CDK12i). A multicenter retrospective study (223) involving 52 cases of CDK12-mutated PCa (including 27 cases with biallelic mutations) reported a median post-metastasis survival of 3.9 years during follow-up, with 63% of patients progressing to CRPC within three years. Additionally, 79% of patients exhibited a tandem duplication signature, and those with a higher degree of tandem duplication dispersion had worse survival outcomes (HR = 2.8, P = 0.01). In contrast, a prospective study (172) found that most patients with biallelic inactivation of CDK12 did not respond to treatment.

Third, the interplay between the TME and immunotherapy remains incompletely characterized, impeding rational design of combinations. As a “cold” tumor, PCa responds poorly to PD-1/PD-L1 inhibitors largely due to an immunosuppressive TME. This suppression reflects the coordinated actions of Tregs, MDSCs, and inhibitory cytokines (e.g., IL-10, TGF-β), which collectively blunt T cell–mediated antitumor immunity. Systematic mapping of stromal–immune crosstalk using single-cell sequencing and organoid co-culture models would advance mechanistic understanding. Improved drug-delivery systems (224) capable of overcoming microenvironmental barriers may enhance the effectiveness of combined ADT, radiotherapy, and chemotherapy.

Fourth, a more comprehensive biomarker system is needed to improve treatment selection. PD-L1 expression and TMB have limited predictive value in PCa (174). Liquid-biopsy assessment of PD-L1/AR-V7 co-expression in CTCs (225) provides real-time readouts of immune-evasion dynamics. Circulating microbiome DNA (cmDNA) (226), a bone metastasis-related gene prognostic index derived from single-cell sequencing of CTCs (227), and blood-based TMB assays (228) all show promise in distinguishing patients from healthy individuals, predicting immunotherapy benefit and durability, and complementing dynamic monitoring of circulating tumor DNA (ctDNA) mutation profiles. Liquid biopsy facilitates dynamic monitoring in PCa immunotherapy through several key mechanisms: firstly, by tracking immune checkpoint expression and evasion mechanisms:pyg, as the shift from PD-L1 to B7-H3 dominance on CTCs following PD-1 inhibitor therapy, indicating adaptive resistance and the need for target adjustment (81); ctDNA-based detection of MSI-H status also demonstrates high concordance with tissue testing (86% sensitivity, 99.5% specificity), supporting patient selection for immune checkpoint inhibitors (229). Secondly, it captures genomic heterogeneity and the evolution of resistant clones; serial next-generation sequencing can trace temporal changes in resistant populations (230). Thirdly, it assists in treatment response prediction and prognostic stratification: AR-V7 positivity in CTCs is associated with significantly shorter progression-free survival under AR pathway inhibitor treatment (1.4 vs. 6.0 months) (231), predicts resistance to AR-targeted therapies (232), and blood-based MSI-H status is significantly correlated with progression-free survival in advanced disease (228). Nevertheless, liquid biopsy remains constrained by insufficient assay standardization, limited prospective validation, and high costs. An integrated model combining genomic markers, immune microenvironment profiling, and metabolomic signatures could yield a quantifiable system for therapeutic-outcome prediction.

Fifth, multi-center studies incorporating population-specific data are needed to generate robust, generalizable evidence. Global initiatives (e.g., CheckMate 650, KEYNOTE-365) have begun addressing racial and regional disparities via multinational collaboration. Establishing STROBE-compliant real-world databases with standardized RECIST v1.1 + PCWG3 composite criteria and interoperable data-sharing platforms would raise research quality. Particular attention should focus on TME dynamics during castration resistance, with adaptive trial designs used to validate novel biomarkers. Ultimately, advances in PCa immunotherapy will require integration of mechanistic discovery, biomarker development, and clinical validation to elevate immunotherapy from a supplementary option to a cornerstone of precision oncology, thereby improving survival outcomes.

## 7 Summary

Although prostate cancer is generally classified as an immunologically “cold” tumor demonstrating a poor response to conventional immunotherapeutic approaches, PD-1/PD-L1 inhibitors manifest significant antitumor activity through three distinct mechanisms: (1) blockade of T-cell inhibitory signaling pathways, (2) enhancement of T-cell activation, and (3) reduction of tumor immune evasion. Nevertheless, the clinical efficacy of PD-1/PD-L1 inhibitor monotherapy in prostate cancer patients persists at suboptimal levels, which may be partially explained by the characteristically low PD-L1 expression levels observed in prostate cancer. Combined therapeutic approaches appear more promising for enhancing patient outcomes. Further research should elucidate the underlying immunotherapeutic mechanisms in PCa and explore innovative treatment modalities. Subsequent investigations must focus on refining the efficacy-to-safety ratio of immunotherapy while evaluating its synergistic potential with existing therapies to optimize clinical results.
